# Supplementary material for: Secondary-structure switch regulates the substrate binding of a YopJ family acetyltransferase
Source: Nat Commun. 2021 Oct 13;12:5969. doi: 10.1038/s41467-021-26183-1 (PMC8514532; doi:10.1038/s41467-021-26183-1)
Supplement: Supplementary file 1 — Supplementary Information [file 41467_2021_26183_MOESM1_ESM.pdf]

Supplementary Information

Secondary-structure switch regulates the substrate binding of a YopJ family acetyltransferase

Yao Xia[1,2,8], Rongfeng Zou[3,8], Maxime Escouboué[4,8], Liang Zhong[1,2,8], Chengjun Zhu[1,2], Cécile Pouzet[5], Xueqiang Wu[6], Yongjin Wang[1,2], Guohua Lv[7], Haibo Zhou[6], Pinghua Sun[1,2*], Ke Ding[1,2*], Laurent Deslandes[4*], Shuguang Yuan[3*], Zhi-Min Zhang[1,2*]

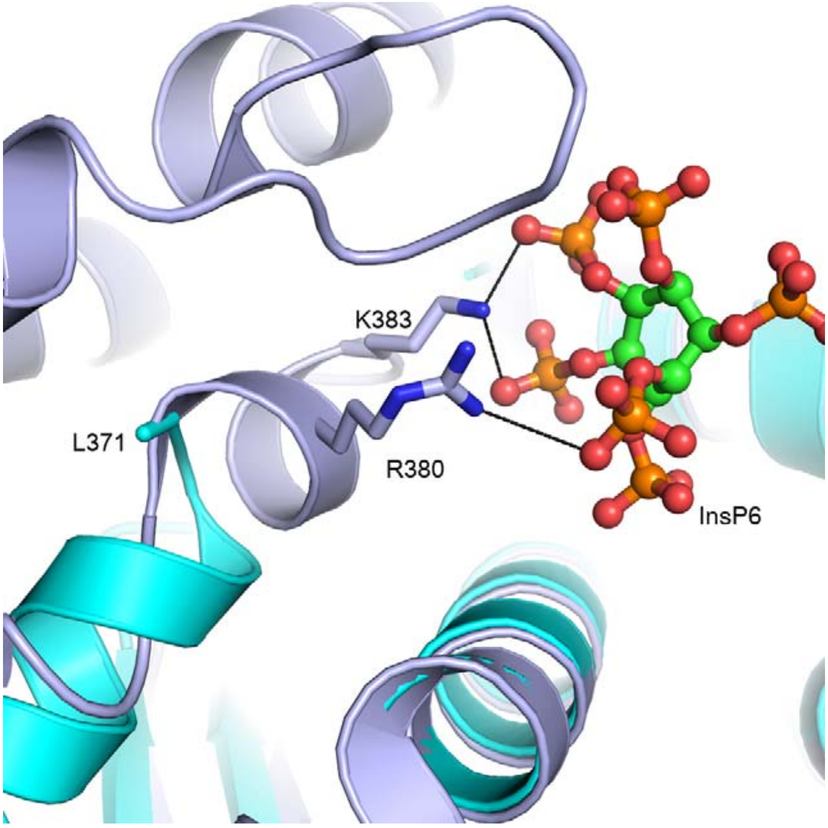

Supplementary Figure 1. Structural comparison of apo-PopP2 and InsP6-bound PopP2 in the InsP6 binding pocket. The structure of apo-PopP2 is colored in cyan and the InsP6-bound in light blue. The InsP6 molecule is shown in stick-and-ball model. The hydrogen-bonding interactions are shown as dashed lines.

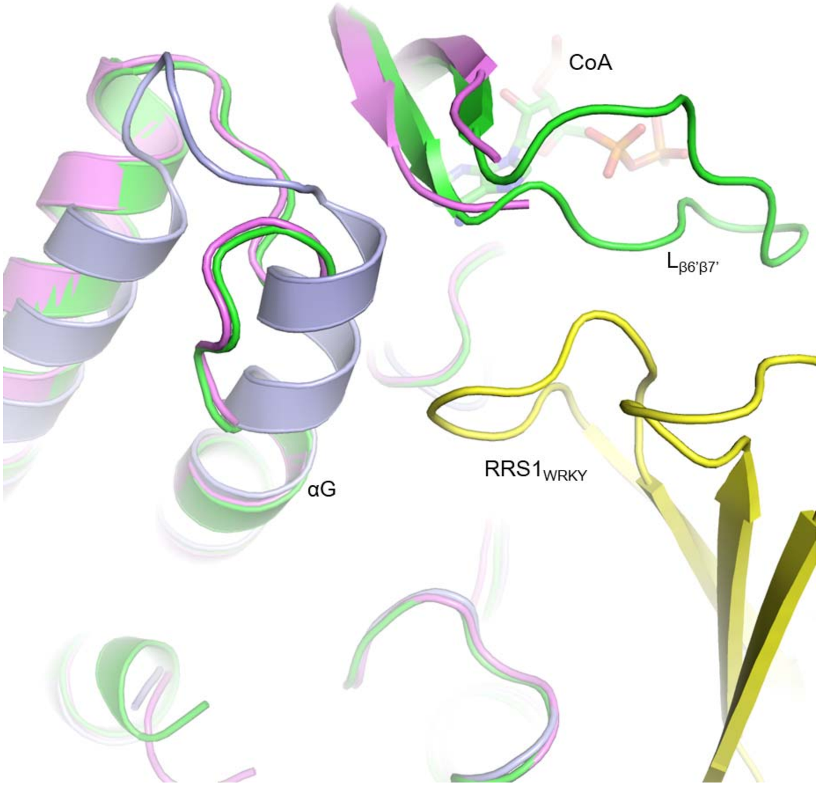

Supplementary Figure 2. Structural comparison of apo-PopP2 (light blue), InsP6-bound PopP2 (pink) and PopP2-InsP6-CoA-RRS1$_{WRKY}$ complex (green) around helix αG. RRS1$_{WRKY}$ is colored in yellow. The CoA molecule is shown in stick-and-ball model.

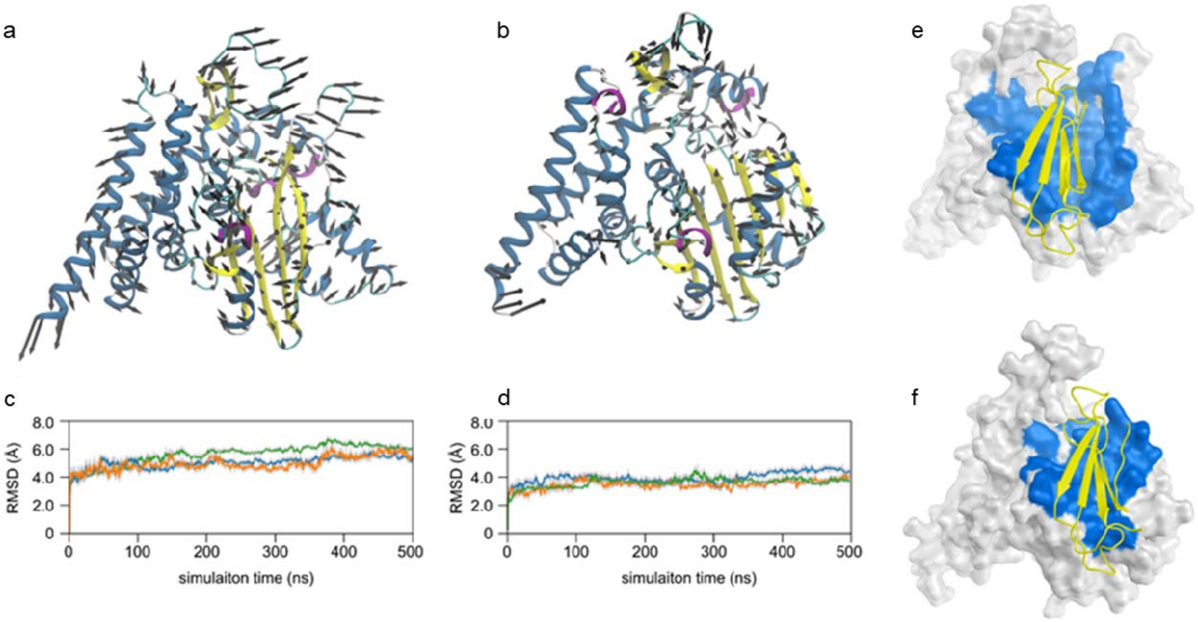

Supplementary Figure 3. The flexibility study of PopP2 in apo (a, c, e) form and in complex with InsP6 (b, d, f). (a) and (b): PCA analysis of PopP2. The length of arrow is correlated to the flexibility of corresponding region. (c) and (d): the RMSD of PopP2 during all-atom MD simulations. Each simulation was repeated three times which were represented by green, blue and orange lines. (e) and (f): the binding pocket surface of PopP2.

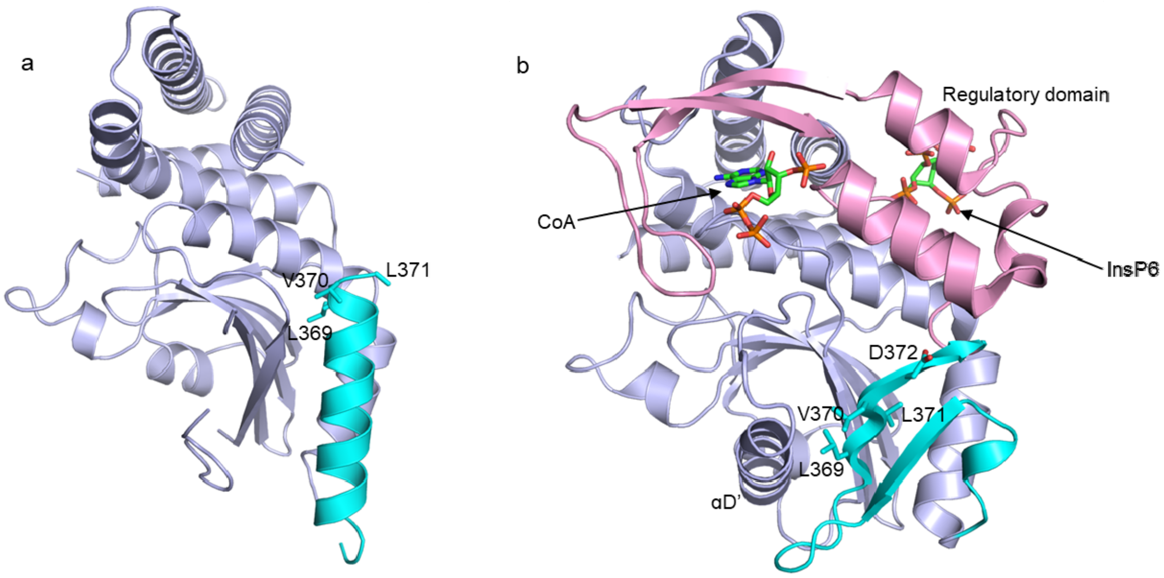

Supplementary Figure 4. Residues selected for mutation study in the fold-switching motif. Fold-switching motif in apo (a) and InsP6-bound (b) structures are colored in cyan, with the side chains of the residues selected for mutation study are shown as sticks.

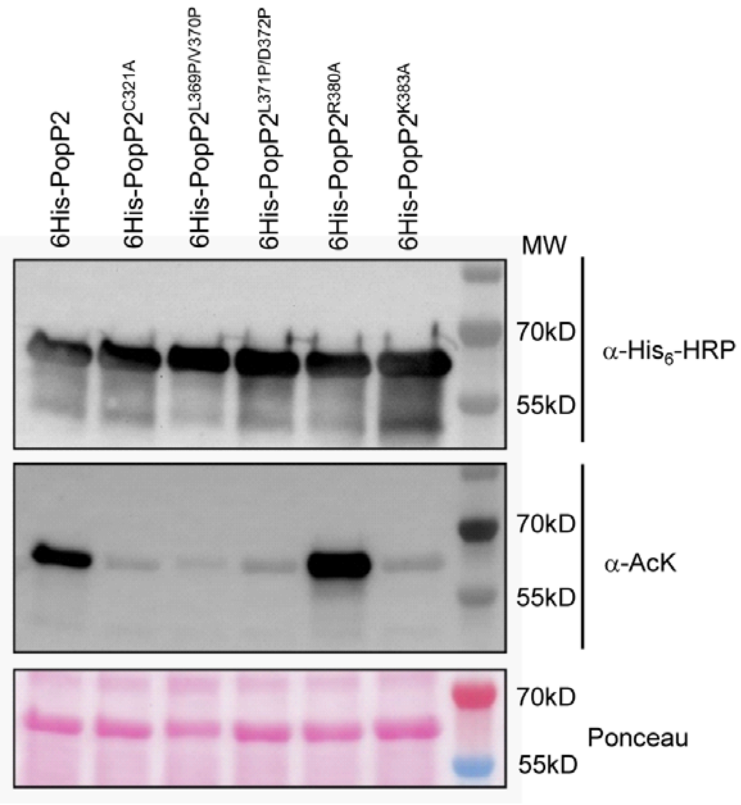

Supplementary Figure 5. PopP2 mutants L369P/V370P, L371P/D372P and K383A are affected in their autoacetylation activity. Recognition of acetylated wild-type PopP2 and R380A mutant, but not PopP2-C321A, PopP2-L369P/V370P, PopP2-L371P/D372P or PopP2-K383A, by an anti-acetyl-lysine antibody. The different PopP2 variants were N-terminally tagged with a 6xhistidine and expressed in Rosetta cells. Recombinant proteins were analyzed by immunoblot using an antibody that recognizes 6xhistidine tagged proteins (α-His6-HRP) or acetylated lysines (α-AcK). 6his-tagged PopP2 proteins are shown after Ponceau staining (top). This experiment was conducted three times with similar results.

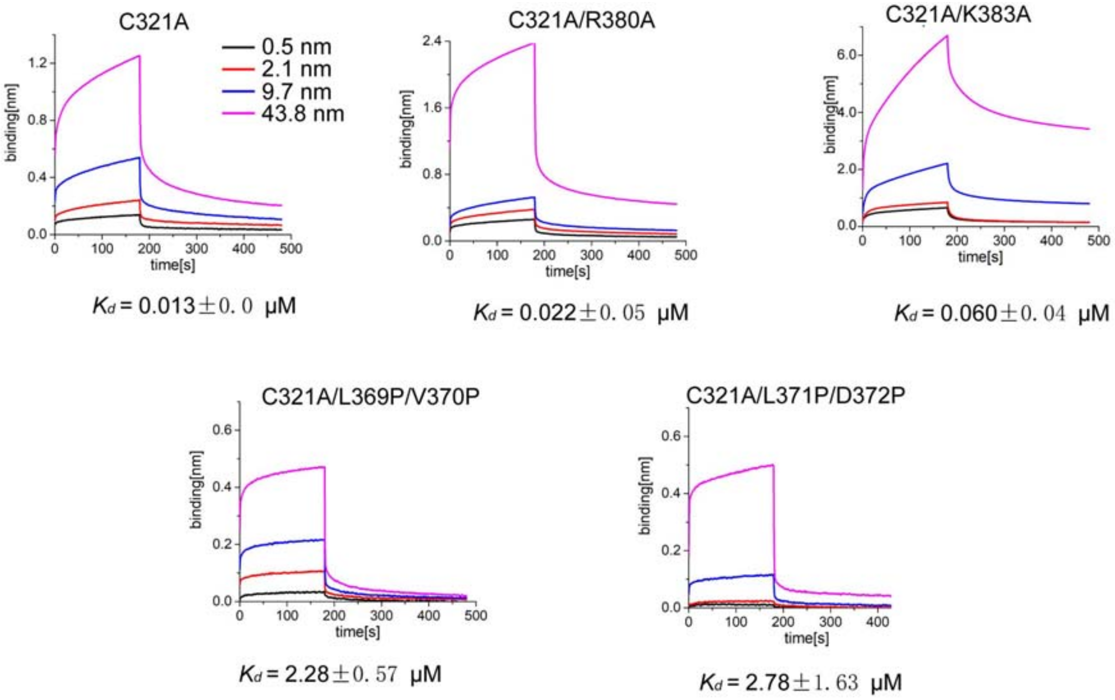

Supplementary Figure 6. Representative binding curves of PopP2 variants to RRS1$_{WRKY}$ in the presence of InsP6 examined by BLI assays. The concentrations of PopP2 variants are labeled in the first panel. Representative curves are from one of two independent experiments.

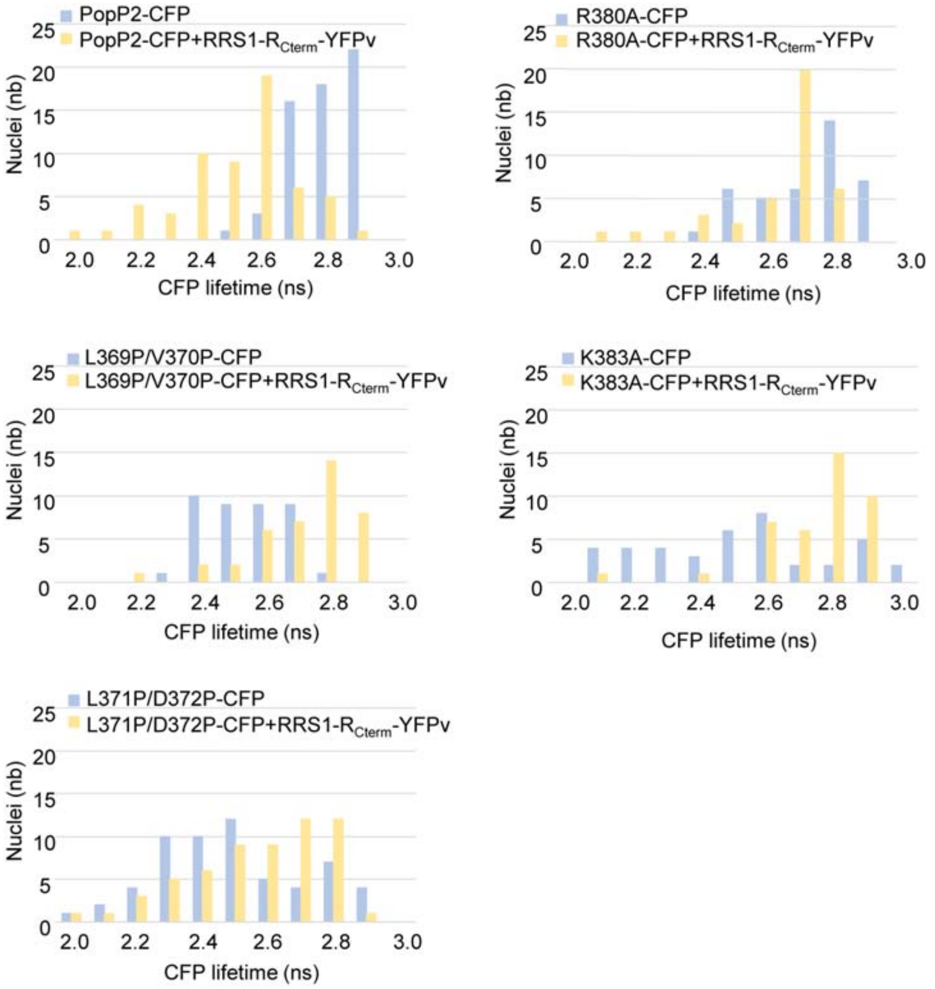

Supplementary Figure 7. PopP2 L369P/V370P, L371P/D372P and K383A mutants are unable to physically interact *in planta* with the C-terminal portion of RRS1-R containing the WRKY domain (RRS1-R$_{Cterm}$). CFP lifetime distribution of the different PopP2 variants fused with CFP and expressed either alone or with RRS1-R$_{Cterm}$-YFPv. Histograms shows the distribution of nuclei (number) according to PopP2-CFP lifetime classes in the absence (blue bars) or presence (orange bars) of RRS1-R$_{Cterm}$-YFPv. The scanned nuclei correspond to those used for FRET-FLIM measurements presented in Table1. This experiment was conducted twice with similar results.

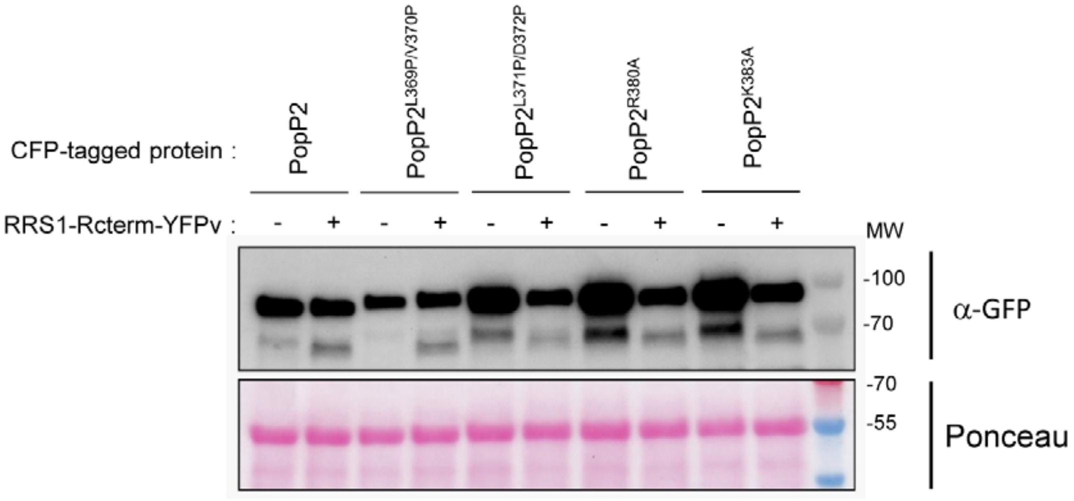

Supplementary Figure 8. Immuno-detection of the different PopP2 variants fused to CFP and expressed either alone or with RRS1-R$_{Cterm}$-YFPv in *N. benthamiana.* The CFP- and YFPv-tagged proteins (crude extracts) are detected with an anti-GFP antibody. Ponceau S staining of total protein demonstrates equal loading of the samples. The star indicates the signal corresponding to RRS1-R$_{Cterm}$-YFPv fusion protein. This experiment was conducted twice with similar results.

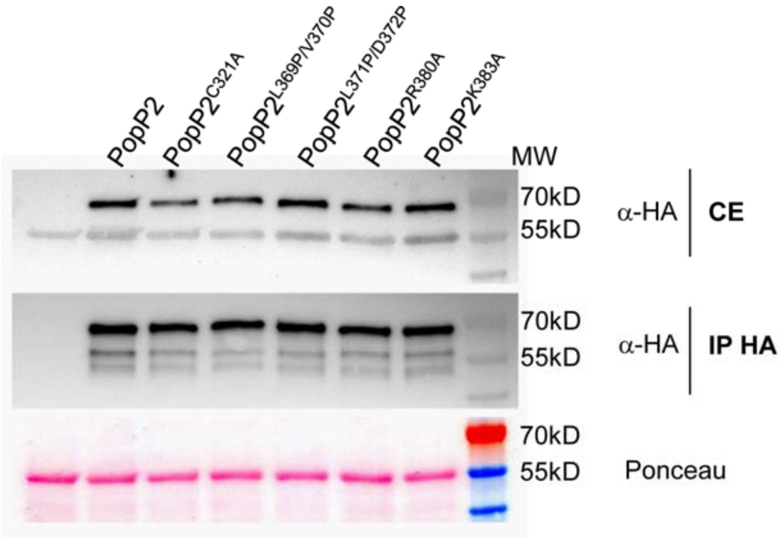

Supplementary Figure 9. **Immuno-detection of the different PopP2 variants delivered by *Pf0-1* in the *rrs1-1* null mutant.** Leaf samples were harvested 7 hours post infiltration (hpi). HA-tagged proteins were detected using an anti-HA-HRP antibody (from crude extracts (CE) and immunoprecipitated proteins). Ponceau staining indicates similar protein amounts loaded in the different lanes. This experiment was conducted three times with similar results.

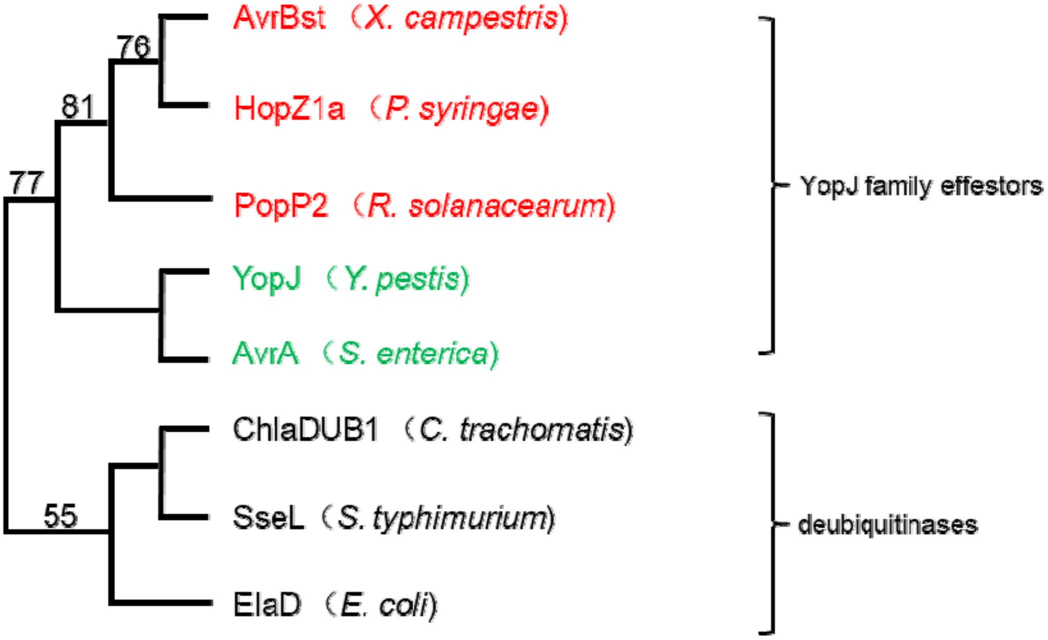

Supplementary Figure 10. Phylogeny of representative CE clan of proteases. The phylogenetic tree was generated with MEGA7 using the sequences of catalytic regions. The GenBank accession numbers of effectors used for this analysis are as follows: AAD39255 for AvrBsT (Residues 61-350), AAR02168 for HopZ1a (Residues 31-369), CAD14570 for PopP2 (Residues 151-488), AKN09807 for YopJ (Residues 1-288), AAL21745 for AvrA (Residues 1-288), B0B9A0.1 for ChlaDUB (Residues 150-401), Q8ZNG2.2 for SseL (Residues159-340) and Q8XCY9.1 for ElaD (Residues 201-407). YopJ effectors produced by plant and animal pathogens are colored in red and green, respectively.

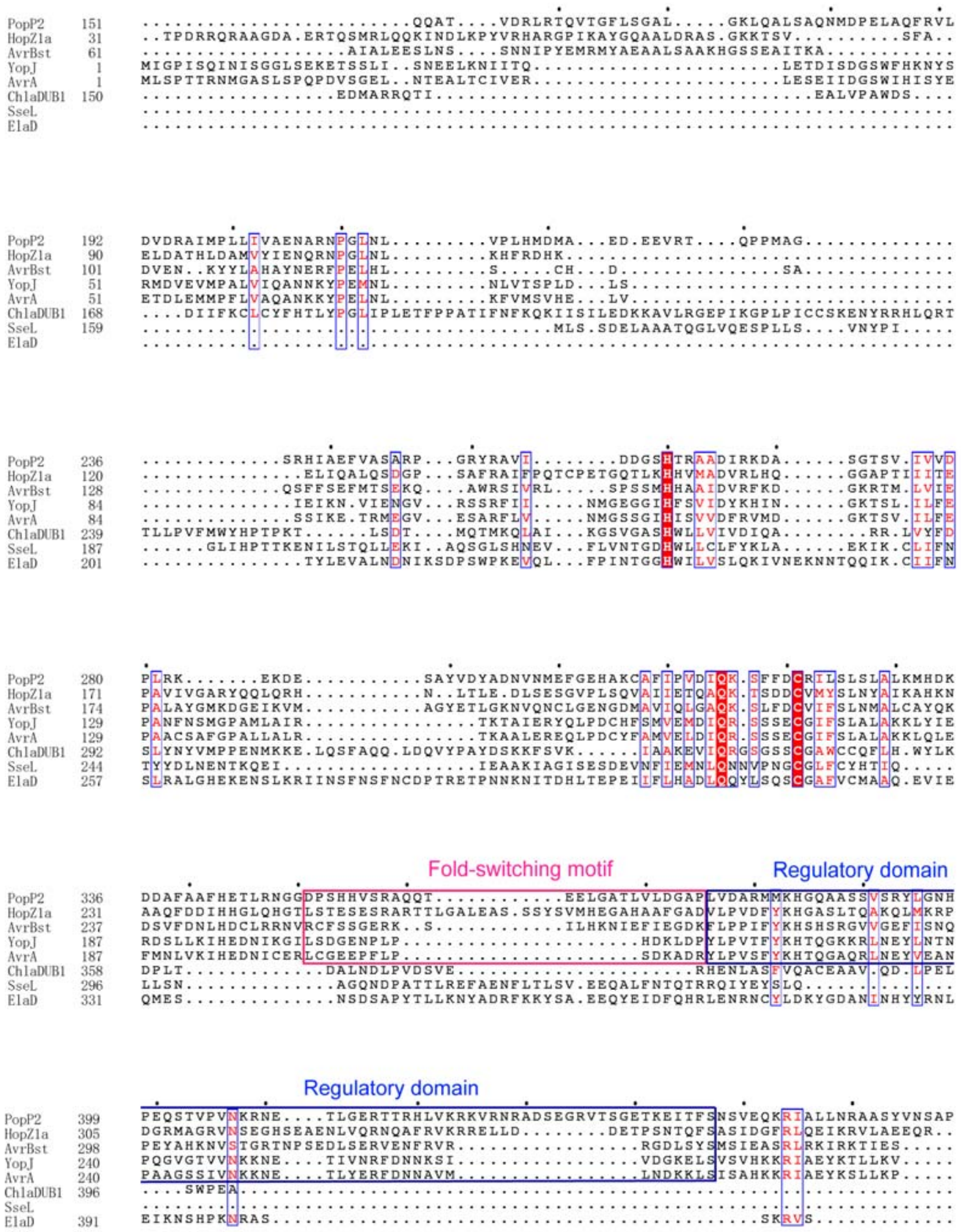

Supplementary Figure 11. Sequence alignment of representative CE clan of proteases. The same sequences were used to perform phylogenetic analysis and sequence alignment. The fold switching motif and regulatory domain are marked by red and blue boxes, respectively.

Supplementary Table 1. Data collection and refinement statistics

	apo-PopP2 (7F3N)
**Data collection**	
Space group	P 3_2 2 1
Cell dimensions	
a, *b*, *c* (Å)	89.4,89.4, 79.9
α, β, γ (°)	90.00, 90.00,120.00
Resolution (Å)	50.00-2.35(2.43-2.35)[a]
R_{merge}	0.136(0.702)
$I/\sigma(I)$	15.9(2.6)
$CC_{1/2}$	0.984(0.898)
Completeness (%)	100.0(100.0)
Redundancy	9.9(10.2)
**Refinement**	
Resolution (Å)	50.00-2.35
No. reflections	15654
R_{work} / R_{free}	0.193/0.231
No. atoms	
Protein	1853
Water	34
B factors	
Protein	68.0
Water	55.4
R.m.s.deviations	
Bond lengths (Å)	0.004
Bond angles (°)	0.78

[a]Values in parentheses are for highest-resolution shell.

Supplementary Table 2. Energies of $RRS1_{WRKY}$ binding to apo- and InsP6-bound PopP2. All terms are in kcal/mol.

	ΔG (kcal/mol)	ΔH (kcal/mol)	-TΔS (kcal/mol)
apo-PopP2	-3.8 ± 3.8	-59.7 ± 5.9	55.9 ± 8.7
InsP6-bound PopP2	-9.1 ± 4.3	-85.3 ± 8.1	76.2 ± 10.5

Supplementary Table 3. Primers used in this study.

Primer name	sequence
AttB1-PopP2	GGGGACAAGTTTGTACAAAAAAGCAGGCTTAATGAAGGTCAGTAGCGCA
AttB2-PopP2	GGGGACCACTTTGTACAAGAAAGCTGGGTCGTTGGTATCCAATAGGGAAT
PopP2-Fw	ATGAAGGTCAGTAGCGCAAACGC
PopP2-Rev	GTCGTTGGTATCCAATAGGGA
PopP2-C321A-Fw	TCCTTCTTCGATGCCCGGATACTCTCCCTGTCACT
PopP2-C321A-Rev	GAGAGTATCCGGGCATCGAAGAAGGACTTCTGAAT
P2-L369-V370P-Fw	AGGAACTTGGCGCTACCCCACCGCTTGATGGTGCGCCACTGGT
P2-L369-V370P-Rev	TGGCGCACCATCAAGCGGTGGGGTAGCGCCAAGTTCCTCCGT
P2-L371-D372P-Fw	GGCGCTACCCTTGTGCCTCCAGGTGCGCCACTGGTCGA
P2-L371-D372P-Rev	ACCAGTGGCGCACCTGGAGGCACAAGGGTAGCGCCAAGT
P2-R380A-Fw	TGGTCGACGCCGCTATGATGAAACATGGTCAAGCCGCA
P2-R380A-Rev	ACCATGTTTCATCATAGCGGCGTCGACCAGTGGCGCAC
P2-K383A-Fwd	GTATGATGGCACATGGTCAAGCCGCA
P2-K383A-Rev	CTTGACCATGTGCCATCATACGGGCGT
