# Supplementary material for: Secondary-structure switch regulates the substrate binding of a YopJ family acetyltransferase
Source: Nat Commun. 2021 Oct 13;12:5969. doi: 10.1038/s41467-021-26183-1 (PMC8514532; doi:10.1038/s41467-021-26183-1)
Supplement: Supplementary file 3 — Description of Additional Supplementary Files [file 41467_2021_26183_MOESM3_ESM.pdf]

## Description of Additional Supplementary Files

**Supplementary Movie 1**. Transition of the fold-switching motif from αhelix into β-strand

**Supplementary Movie 2**. Structural changes of PopP2 without the presence of InsP6

**Supplementary Movie 3**. Structural changes of PopP2 in the presence of InsP6

**Supplementary Movie 4**. An example trajectory in the metadynamics simulations
