# Supplementary material for: Secondary-structure switch regulates the substrate binding of a YopJ family acetyltransferase
Source: Nat Commun. 2021 Oct 13;12:5969. doi: 10.1038/s41467-021-26183-1 (PMC8514532; doi:10.1038/s41467-021-26183-1)
Supplement: Supplementary file 8 — Reporting summary [file 41467_2021_26183_MOESM8_ESM.pdf]

nature portfolio

Corresponding author(s): ZHI-MIN ZHANG
13632107756@163.com

Last updated by author(s): Sep 11, 2021

Reporting Summary

Nature Portfolio wishes to improve the reproducibility of the work that we publish. This form provides structure for consistency and transparency in reporting. For further information on Nature Portfolio policies, see our Editorial Policies and the Editorial Policy Checklist.

Statistics

For all statistical analyses, confirm that the following items are present in the figure legend, table legend, main text, or Methods section.

n/a	Confirmed	
☐	☒	The exact sample size (*n*) for each experimental group/condition, given as a discrete number and unit of measurement
☐	☒	A statement on whether measurements were taken from distinct samples or whether the same sample was measured repeatedly
☐	☒	The statistical test(s) used AND whether they are one- or two-sided<br>*Only common tests should be described solely by name; describe more complex techniques in the Methods section.*
☒	☐	A description of all covariates tested
☒	☐	A description of any assumptions or corrections, such as tests of normality and adjustment for multiple comparisons
☐	☒	A full description of the statistical parameters including central tendency (e.g. means) or other basic estimates (e.g. regression coefficient) AND variation (e.g. standard deviation) or associated estimates of uncertainty (e.g. confidence intervals)
☐	☒	For null hypothesis testing, the test statistic (e.g. *F*, *t*, *r*) with confidence intervals, effect sizes, degrees of freedom and *P* value noted<br>*Give P values as exact values whenever suitable.*
☒	☐	For Bayesian analysis, information on the choice of priors and Markov chain Monte Carlo settings
☒	☐	For hierarchical and complex designs, identification of the appropriate level for tests and full reporting of outcomes
☒	☐	Estimates of effect sizes (e.g. Cohen's *d*, Pearson's *r*), indicating how they were calculated

Our web collection on statistics for biologists contains articles on many of the points above.

Software and code

Policy information about availability of computer code

Data collection	X-ray diffraction data were collected at the on the beam-line BL19U1 at the Shanghai Synchrotron Radiation Facility.
Data analysis	For structural study, the HKL2000, XDS, PHENIX v1.16_3549-000, Coot v0.8.9 and Pymol v0.99 softwares were used for data processing and analysis. Computational studies were performed with GROMACS-2018.4, and Protein Preparation Wizard and Prime in Schrödinger software. AMBER18 was used to obtain the trajectories as well as the MM/GBSA calculations

For manuscripts utilizing custom algorithms or software that are central to the research but not yet described in published literature, software must be made available to editors and reviewers. We strongly encourage code deposition in a community repository (e.g. GitHub). See the Nature Portfolio guidelines for submitting code & software for further information.

Data

Policy information about availability of data

All manuscripts must include a data availability statement. This statement should provide the following information, where applicable:
- Accession codes, unique identifiers, or web links for publicly available datasets
- A description of any restrictions on data availability
- For clinical datasets or third party data, please ensure that the statement adheres to our policy

The authors declare that all data supporting the findings of this study are available within the article [and its supplementary information files]. The crystal structure has been deposited in Protein Data Bank under the code 7F3N.

Field-specific reporting

Please select the one below that is the best fit for your research. If you are not sure, read the appropriate sections before making your selection.

- [x] Life sciences
- [ ] Behavioural & social sciences
- [ ] Ecological, evolutionary & environmental sciences

For a reference copy of the document with all sections, see nature.com/documents/nr-reporting-summary-flat.pdf

Life sciences study design

All studies must disclose on these points even when the disclosure is negative.

Sample size	Biochemical and enzymatic assays were completed using wild type or mutants of PopP2 fragments. All experiments performed in planta were conducted using full length PopP2 variants. The sample size is sufficient to delineate the mutational effects of PopP2.
Data exclusions	No data excluded
Replication	The in vitro acetylation and BLI assays were performed twice with similar result. PopP2-triggered cell death FRET- assays were performed three times as stated in figure legends. FRET-FLIM measurements were performed from at least 2 independent transient assays. In planta WRKY acetylation assays and PopP2 autoacetylation in E. coli were conducted three times independently.
Randomization	The assays performed in this study require a rational approach for activity comparison. Therefore, randomization is not applicable to our experimental set up
Blinding	Blinding is not applicable to any biochemical or cellular assay performed in this study.

Reporting for specific materials, systems and methods

We require information from authors about some types of materials, experimental systems and methods used in many studies. Here, indicate whether each material, system or method listed is relevant to your study. If you are not sure if a list item applies to your research, read the appropriate section before selecting a response.

Materials & experimental systems

n/a	Involved in the study	
☐	☒	Antibodies
☒	☐	Eukaryotic cell lines
☒	☐	Palaeontology and archaeology
☒	☐	Animals and other organisms
☒	☐	Human research participants
☒	☐	Clinical data
☒	☐	Dual use research of concern

Methods

n/a	Involved in the study	
☒	☐	ChIP-seq
☒	☐	Flow cytometry
☒	☐	MRI-based neuroimaging

Antibodies

Antibodies used	- Anti-Ac-Lysine antibody (AKL5C1, sc32268, Santa Cruz Biotech) ; Anti-Ac-Lysine antibody (Mouse mAb, Ac-K-103, Cell Signaling)<br>- Anti-GFP (from mouse IgG1κ (clones 7.1 and 13.1)) from ROCHE<br>- Goat anti Mouse IgG (H+L)-HRP conjugate (Bio-Rad, #1706516)<br>- Goat anti-Mouse IgG2a-HRP conjugate (Bio-Rad, STAR133P)<br>- Anti-His6-peroxydase (BMG-his-1, mouse monoclonal IgG1, ref. 11 965 085 001) from ROCHE
Validation	- Anti-Ac-lysine Antibody (AKL5C1) is a mouse monoclonal IgG1 κ Ac-lysine antibody, cited in 52 publications. Ac-lysine Antibody (AKL5C1) is recommended for detection of proteins containing N-epsilon-acetylated lysine residues by WB, IP, IF and IHC(P); non cross-reactive with N-α-acetylated lysine (https://www.scbt.com/p/ac-lysine-antibody-akl5c1?requestFrom=search).<br>- Anti-Acetylated Lysine Antibody (Ac-K-103) is a mouse monoclonal, cited in 122 publications. (Ac-K-103) is recommended for detection of proteins post-translationally modified by acetylation on the epsilon-amine groups of lysine residues (Application : WB, IP, IHC, ChIP, IF, F and E-P) (https://www.cellsignal.com/products/primary-antibodies/acetylated-lysine-mouse-mab-ac-k-103/9681).<br>- Anti-GFP (from mouse IgG1κ (clones 7.1 and 13.1)) is a mixture of two high-affinity monoclonal antibodies selected for their performance in detection of GFP and GFP fusion proteins (Application : IP, WB, Immunostaining).<br>- Goat anti-Mouse IgG (H+L)-HRP conjugate (Bio-Rad) recognizes primary monoclonal antibodies from Mouse.<br>- Goat anti-Mouse IgG2a-HRP conjugate (Bio-Rad, STAR133P) is a polyclonal antibody that recognizes Mouse IgG2a (Application : Immunohistology, Elisa, WB). Used in teh study for the detection of primary Ac-K-103 Mouse mAb.<br>- Anti-His6-peroxydase (BMG-his-1, mouse monoclonal IgG1) specifically recognizes an epitope of six consecutive histidine residues of both natural and recombinant proteins (Application : Elisa, WB).
